# UAV LiDAR point cloud and ground measurement dataset for assessing the impact of flight parameters over a poplar plantation

**DOI:** 10.1016/j.dib.2026.113234

**Published:** 2026-09-02

**Authors:** Frédéric Baup, Clément Battista, Frédéric Frappart

**Affiliations:** a CNES/IRD/CNRS/INRAe, CESBIO, University of Toulouse, 31000 Toulouse, France; b INRAE, Bordeaux Science Agro, UMR ISPA, 33140 Villenave d’Ornon, France

**Keywords:** UAV LiDAR, Tree-level metrics, Canopy height, Trunk circumference, Flight configuration, Plantation forestry

## Abstract

This data article presents a UAV LiDAR dataset acquired over a 0.23-ha managed poplar (*Populus* spp.) plantation located in southwestern France. The dataset was designed to assess the influence of UAV flight parameters and acquisition context on the extraction of tree-level structural metrics, including canopy height and trunk circumference. A total of 22 UAV LiDAR flights were conducted between January 2024 and April 2025 using a DJI Matrice 300 platform equipped with a Zenmuse L1 sensor. Acquisition parameters were systematically varied, including flight altitude (35–120 m above ground level), flight speed (1.7–6 m·s⁻¹), return mode (single, dual, triple), flight plan orientation relative to plantation rows (0°, 45°, 90°), overlap margins, and seasonal conditions (leaf-off and leaf-on). Resulting point densities range from 447 to 12,990 points·m⁻². Raw LiDAR data were processed into georeferenced LAS point clouds (Lambert-93, EPSG:2154; EGM96 geoid). The dataset includes several complementary data products supporting the evaluation of UAV LiDAR-derived structural metrics. These comprise: (i) canopy height estimates and trunk circumference measurements derived from the UAV LiDAR point clouds using three methods (mean circle fitting, maximum inscribed circle fitting, and spline fitting); (ii) *in situ* trunk circumference measurements collected during three field campaigns (December 2024, April 2025, October 2025) and (iii) tree height and trunk circumference measurements derived from terrestrial LiDAR acquisitions (TLS) using a backpack-mounted system (Viametris MS-96).

Specifications Table

**Table:** 

Subject	Earth & Environmental Sciences
Specific subject area	UAV LiDAR forest inventory tree height and trunk circumference estimation in managed poplar plantations
Type of data	Type of data Table; Raster; Point cloud; Metadata fil Data format / processing level Converted; Classified; Segmented; Filtered; Processed; Analyzed; Georeferenced; orthoimages File formats LAS (.las); CSV (.csv); GeoTIFF (.tiff); Geopackage (.gpkg); Txt (.txt);
Data collection	LiDAR data were collected over a 0.23 ha poplar plantation using a DJI Matrice 300 UAV equipped with a Zenmuse L1 LiDAR sensor (905 nm, up to 240 kpts.s-1, single/dual/triple returns, RTK < 5 cm). Data acquisition was acheived during twenty-two flights in 2024 and 2025 at varied altitude (35–120 m above ground), speed, orientation, number of returns, margins, and seasonality (winter and spring, leaf-off and leaf-on). Raw data were converted to LAS using DJI Terra (EPSG: 2154, EGM96). Ground points were classified with ArcGIS. Field circumferences were measured with flexible tapes at 0.60, 1.30, and 2.00 m.
Data source location	The data were collected in a managed poplar (*Populus* spp.) plantation located in Ordan-Larroque (43.690,893° N, 0.464,305° E), Occitanie region, southwestern France. The study area covers 0.23 ha within a 1.4 ha plantation at an average elevation of 149 m A.G.L. (Above Ground Level)
Data accessibility	Repository name:https://entrepot.recherche.data.gouv.fr/dataverse/CESBIO Direct URL to data:https://entrepot.recherche.data.gouv.fr/dataset.xhtml?persistentId=doi:10.57745/WAEYEX Instructions for accessing these data: All processed datasets are publicly available under an Etalab Open License 2.0. The repository contains a README file describing folder structure, file formats, coordinates reference system. Data can be downloaded directly from the repository landing page. No access restrictions apply.
Related research article	C. Battista, F. Frappart, M. Schwartz, F. Baup, “Impact of UAV flight parameters and acquisition context for canopy height and trunk circumference measurement using LiDAR”, In revision, *Annals of Forest Science* in April 2026.

## 1 Value of the Data

• This dataset provides a controlled, multi-annual UAV LiDAR test site acquired over a homogeneous and regularly spaced poplar plantation. The regular plantation geometry and limited structural complexity allow analyses to focus specifically on acquisition parameterization. The dataset includes 22 flights conducted across two years under different seasonal conditions (leaf-off and leaf-on), with systematically varied flight parameters and complete tree-level reference measurements. It combines processed point clouds, derived tree-level metrics, and field data.
• The data enable structured assessment of the influence of UAV flight parameters (altitude above canopy, speed, overlap, flight plan orientation, return mode, margins) on point cloud density, stem visibility, and canopy representation.
• The repeated acquisitions across seasons (winter and spring) and years provide material to examine the role of phenology, understory development, and structural change under consistent plantation geometry and spacing.
• The dataset can support benchmarking and intercomparison of various methods for tree segmentation, stem measurements, and canopy height extraction. It also provides a structured basis for intercomparison of canopy height models (CHMs/DHMs) derived from different platforms and processing workflows. The availability of UAV LiDAR data alongside airborne LiDAR and satellite-derived canopy height products enables comparison and validation exercises across spatial resolutions and sensor types, including products generated from spaceborne observations.
• Tree height and trunk circumference measurements included in the dataset allow testing of allometric modeling workflows and above-ground biomass (AGB) estimation approaches. The availability of structured tree-level metrics supports applications in forestry and forest management requiring standardized structural variables for inventory, monitoring, and quantitative assessments.

## 2 Background

Accurate estimation of tree height and trunk circumference is essential for forest inventory, biomass assessment, and silvicultural monitoring [[1], [2], [3]]. UAV-borne LiDAR enables high-resolution characterization of forest structure and tree-level measurements [[4], [5], [6]], and supports the validation of satellite-derived products [[7], [8]]. Several public UAV datasets already exist, but were designed for other purposes: [9] targets machine-learning-based individual-tree segmentation, whereas the multi-platform dataset of [10] focuses on cross-sensor and multi-season comparison. In these datasets, flight parameters are generally fixed or not varied systematically, so the influence of the acquisition configuration on tree-level metrics cannot be isolated, a question that remains insufficiently documented under controlled conditions, with few exceptions [11]. The originality of the present dataset lies in its controlled acquisition design: flights were acquired over a homogeneous poplar plantation while varying altitude, speed, return mode, flight orientation, overlap, and season, and holding other conditions constant. It provides a dedicated benchmark for quantifying how flight configuration affects canopy height and trunk circumference, complemented by reference measurements.

## 3 Data Description

The dataset is organized into three main folders. A README file is provided at the root level describing folder structure, coordinate reference system, file naming conventions, and metadata fields.

**/01_UAV_LiDAR/.**

Contains the UAV LiDAR point clouds corresponding to the 22 acquisition flights and associated ancillary data.

• YYYYMMDD_UAV_flightnumber_pointdensity_flightaltitude_speed_flightangle_returnmode.las (example: 20,240,124_UAV_flight01_671_120_6_0_dual.las) Full georeferenced point cloud in LAS format (EPSG:2154; EGM96 vertical reference). Density in point.m-2, flight altitude in m, speed in m.s-1, flight angle in degrees to plantation rows.
• UAV_flights_metadata.csv
  Synthetic metadata table including: acquisition date, leaf-on/leaf-off condition, flight altitude, speed, overlap, return mode, flight orientation, and point density.
• UAV_flights_plans.gpkg
  Geopackage file containing flight line geometries of each UAV mission.
• UAV_flights_areas.gpkg
  Geopackage file containing flight polygon AOI of each UAV mission.
• UAV_AOI.gpkg
  Geopackage file containing the area of interest.

**/02_UAV_ORTHO/**

Contains UAV orthophotography products derived from RGB imagery.

• YYYYMMDD_UAV_flightXX_ortho.tiff
  Orthophoto of the study area in GeoTIFF format (georeferenced in EPSG:2154).
• YYYYMMDD_UAV_flight XX_image_position.gpkg
  Position of the UAV sensor when each image of the orthomosaic was taken.
• UAV_ortho_metadata.txt
  Metadata file describing ground sampling distance, acquisition date, processing parameters, and coordinate reference system.

**/03_TREES_METRICS/.**

Contains tree-level field measurements and LiDAR-derived structural metrics.

• trees_metrics.csv
  Tabular dataset including *in situ* circumference measurements (flexible tape) and LiDAR-derived tree metrics (height and trunk circumference). The columns entitled: H_UAV_202,401, H_UAV_202,501, H_TLS_202,501 represents the height estimated from UAV and TLS acquisitions, respectively performed on January 24th 2024 (UAV flight ID 8), January 31th 2025 (UAV flight ID 14) and January 26th 2025 (TLS). The columns named: C_UAV_202,401, C_MAN_202,412, C_TLS_202,501, represent the trunk circumferences respectively estimated from UAV flight on January 24th 2024 (flight ID 8), manually performed on 1st December 2024 and estimated from terrestrial LiDAR acquisitions on 26th January 2025. If required, DBH values can be easily derived from the trunk circumference measurements provided in the dataset by dividing the circumference by π.
• trees_metrics.gpkg
  Georeferenced version (EPSG:2154) of the same dataset in GeoPackage format, including precise tree locations and associated attributes (coming from the file trees_metrics.csv).

## 4 Experimental Design, Materials and Methods

### 4.1 Study site configuration

**Fig. 1:**
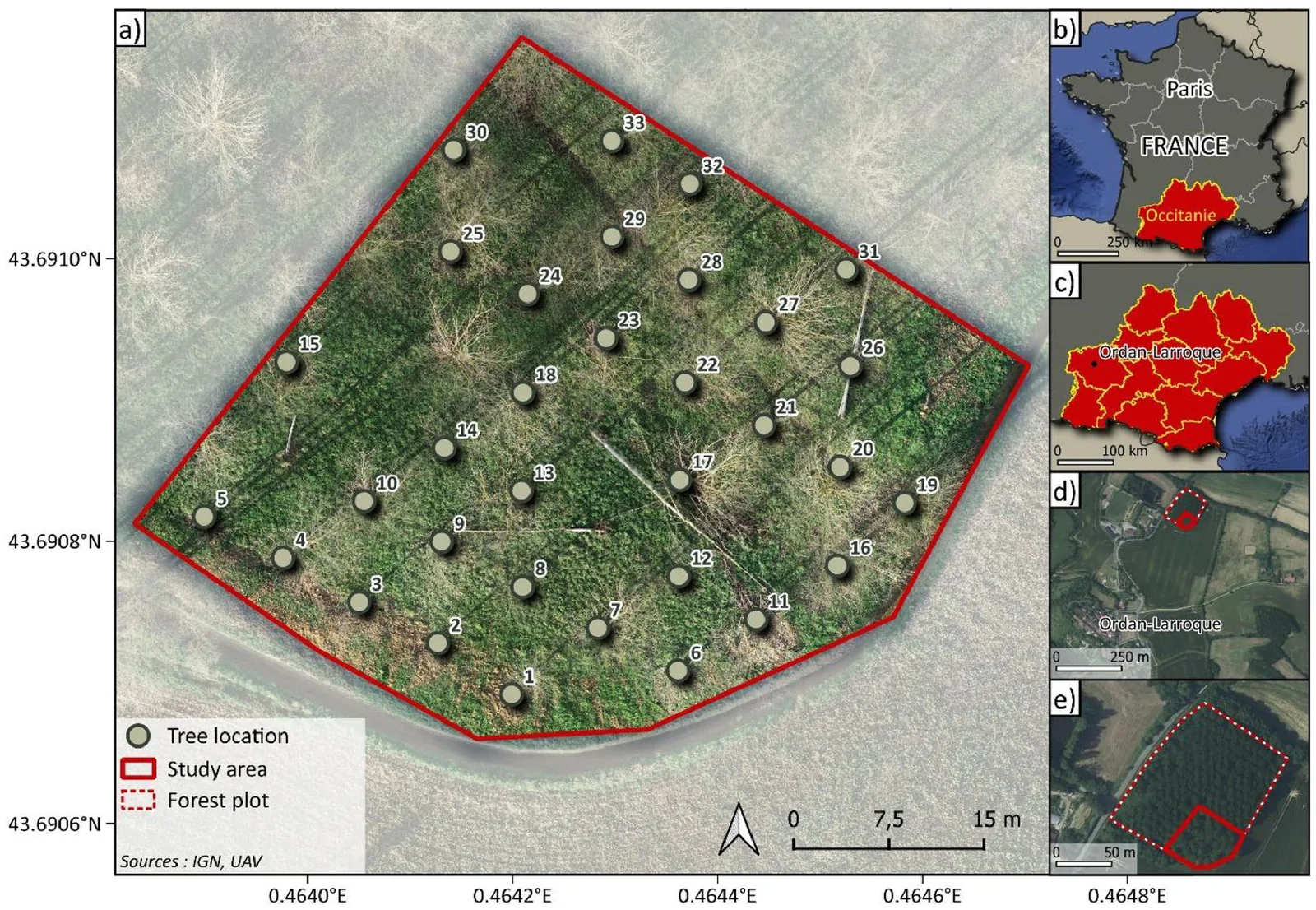
Overview of the trees and study area (a), showing their location from the national scale to the forest plot scale (b-e). The 2024 UAV orthomosaic is displayed in (a), while a 2025 aerial orthomosaic from IGN is shown in (d) and (e).

Data were acquired over a 0.23 ha subsection of a managed poplar (*Populus* spp.) plantation located in Ordan-Larroque, Occitanie, France (43.690,893° N, 0.464,305° E, see Fig. 1). Trees are planted in regular rows oriented northwest–southeast with approximately 7 m spacing within rows and 8 m between rows. Terrain slope is approximately 1%. Elevation is ∼149 m above sea level.

### 4.2 UAV LiDAR data acquisition.

– UAV platform and sensor.

UAV flights were conducted using:

• Platform: DJI Matrice 300 RTK
• LiDAR sensor: DJI Zenmuse L1
  ○ Wavelength: 905 nm
  ○ Maximum pulse rate: 240,000 pts·s⁻¹
  ○ Return modes: single, dual, triple
  ○ Ranging accuracy: 3 cm at 100 m
  ○ Integrated IMU and GNSS
• Positioning: RTK-enabled GNSS (<5 cm planimetric and vertical accuracy)
• RGB camera: 20 MP (used for orthophoto generation)
– Flight design

Twenty-two flights were conducted between January 2024 and April 2025. The following parameters were systematically varied (Table 1):

• Flight altitude: 35–120 m above ground level
• Flight speed: 1.7–6 m·s⁻¹
• Return mode: single, dual, triple
• Flight line orientation: 0°, 45°, 90° relative to plantation rows
• Longitudinal and lateral margins (buffer around the area of interest: 17m for all flight except for flight 11, 12 and 13 fog which the marge have been set to 0m)
• Side OverLap (SOL expressed in percentage)
• Seasonal condition: leaf-off (winter) and leaf-on (spring)

**Table 1:** Table of flights metadata.

Flight number	Date	Density	Flight altitude	GSD	SOL	Distance/ surface covered	Speed	Flight angle	Return mode	Data weight
-	-	Pt.m^-^²	m	cm	%	m (m²)	m.s^-1^	° to plantation rows		Go.Ha^-1^
1	Jan. 2024	671	120	3.27	70	309/4969	6	0	Dual	0.23
2	Jan. 2024	447	120	3.27	70	309/4964	6	0	Triple	0.17
3	Jan. 2024	894	90	2.46	70	300/3838	6	0	Dual	0.40
4	Jan. 2024	596	90	2.46	70	300/3845	6	0	Triple	0.28
5	Jan. 2024	1519	60	1.64	70	610/8738	5.3	0	Dual	1.08
6	Jan. 2024	1013	60	1.64	70	610/8737	5.3	0	Triple	0.80
7	Jan. 2024	2025	45	1.23	70	746/8871	5.3	0	Dual	1.51
8	Jan. 2024	1350	45	1.23	70	746/8881	5.3	0	Triple	1.15
9	Jan. 2024	3838	45	1.23	70	1156/10,442	4	0	Dual	3.10
10	Jan. 2024	1006	80	2.18	70	1234/256,667	6	0	Dual	1.15
11	Jan. 2025	6674	45	1.23	80	2109/19,336	2.3	90	Dual	19.24
12	Jan. 2025	6978	45	1.23	80	2438/22,655	2.2	0	Dual	16.79
13	Jan. 2025	9889	35	0.96	80	2704/20,019	2	90	Dual	5.53
14	Apr. 2025	2683	45	1.23	70	746/8844	4	0	Dual	3.85
15	Apr. 2025	3577	45	1.23	70	746/8780	3	0	Dual	5.07
16	Apr. 2025	5366	45	1.23	70	746/8715	2	0	Dual	7.44
17	Apr. 2025	2683	45	1.23	70	746/8829	4	0	Single	1.21
18	Apr. 2025	1789	45	1.23	70	746/8846	4	0	Triple	1.46
19	Apr. 2025	4386	45	1.23	80	1156/10,409	3.5	0	Dual	6.67
20	Apr. 2025	12,990	45	1.23	85	1454/10,075	1.7	0	Dual	26.79
21	Apr. 2025	2683	45	1.23	70	751/8951	4	45	Dual	1.93
22	Apr. 2025	2683	45	1.23	70	749/8855	4	90	Dual	1.96

**Fig. 2:**
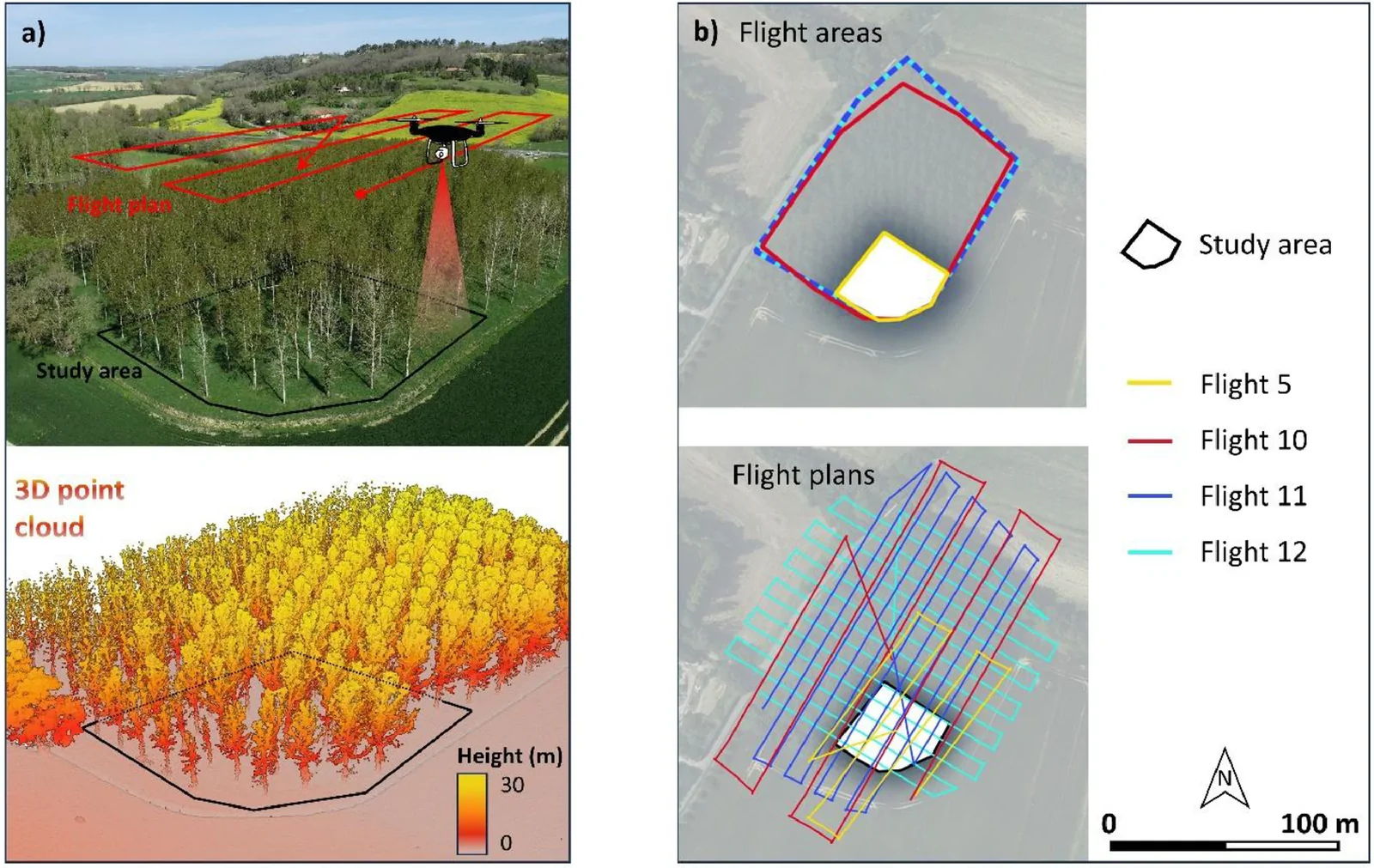
(a) Illustration of the UAV flight planning approach over the study area and the resulting 3D point cloud obtained (imagery acquired in April 2025). (b) Examples of selected flight areas along with their corresponding flight plans generated using DJI software.

Maximum altitude was constrained to 120 m (European UAV regulation). Weather conditions: clear sky, low wind speed (<5 m·s⁻¹). An illustration of the acquisition process is provided in Figure 2.

### 4.3 UAV LiDAR and RGB images data processing

– Conversion and georeferencing

Raw data were recorded in DJI proprietary format and converted to LAS using the free version of DJI Terra. Processing settings:

• Output coordinate reference system: Lambert-93 (EPSG:2154)
• Vertical reference: EGM96 geoid
• Two RGB Orthophotos were generated with Pix4D by collecting and merging all the photos taken by the sensor’s camera, during the flights n°9 (jan. 2024, 1.23 cm GSD) and n°22 (april 2025, 1.23 cm GSD)
– **Ground classification**

Ground points were classified from the LAS point clouds using the Classify LAS Ground tool from ArcGIS Pro 3.6 (3D Analyst toolbox). We used the Standard Classification as the Ground Detection Method and the Latest Detection Algorithm. The Standard Classification is designed to provide robust ground detection across a broad range of terrain conditions, while the Latest algorithm corresponds to the most recent version of the ground-detection procedure implemented in ArcGIS Pro.

### 4.4 LiDAR (UAV and TLS) tree height and circumference estimation methods

Tree height and trunk circumference were extracted from the LiDAR point clouds after individual tree and trunk segmentation. Tree height was estimated from the highest 0.20 m point-cloud slice located above the trunk centroid within a 2 m radius. The median elevation of points within this slice was used as the tree height, rather than the maximum point elevation, to reduce the influence of noise and ensure more robust comparisons between datasets with different point densities and acquisition characteristics. This methods as used to fill the columns: H_UAV_202401, H_UAV_202501, H_TLS_202501 of the file “tree_metrics.csv” (or *.gpkg*)

Trunk circumference was estimated from successive 0.20 m horizontal slices between 0.60 and 2.00 m above ground using a Spline Fitting (SF) method. For each tree, the appropriate cross-sectional representation was selected based on the smallest estimated circumference. Moreover, additional point-cloud filtering was performed to remove branches, basal shoots, ivy, and noise.

The associated research article deeper investigates different methods for tree-level height and trunk circumference estimates from this dataset [12].

### 4.5 Ancillary datasets

Three campaigns of *in situ* trunk circumference measurements were conducted in December 2024, April 2025, and October 2025. Measurements taken with flexible measuring tapes:

• At breast height (1.30 m above ground), following standard DBH (diameter at breast height) protocols [13,14] in December 2024 and April 2025;
• At above ground height of 0.60m, 1.30m, and 2.00m in October 2025.

These field campaigns were conducted by two different operators and using slightly different measuring tools.

## Limitations

The main limitation of this dataset is the relatively small and homogeneous study area, which covers 0.23 ha within a managed poplar plantation characterized by regular tree spacing and low structural complexity. This configuration was deliberately selected to provide a controlled experimental framework in which the effects of UAV LiDAR acquisition parameters could be investigated while minimizing confounding effects related to tree structure. Consequently, the dataset should primarily be considered as a controlled benchmark for assessing the influence of flight configuration and acquisition context on tree-level metrics, rather than as a representation of the diversity of forest ecosystems. The transferability of results obtained from these data to natural or structurally heterogeneous forests, other tree species, different stand densities, complex terrain, or substantially different understory conditions therefore remains to be assessed. In addition, the dataset was acquired using a single UAV platform and LiDAR sensor.

Future datasets should extend this experimental framework to a wider range of tree species, stand structures and densities, terrain conditions, understory characteristics, acquisition platforms and environmental contexts. Synchronous or repeated reference measurements of tree height would also be valuable for future datasets, allowing a better validation of UAV LiDAR-derived height estimates.

## Ethics Statement

The authors have read the ethical requirements for publication in Data in Brief and confirm that the current work does not involve human subjects, animal experiments, or any data collected from social media platforms.

## Credit Author Statement

**Frédéric Baup:** UAV LiDAR and field investigation, data processing, data curation, software, conceptualization, methodology, supervision, writing - review and editing, project administration, funding acquisition. **Clément Battista:** Field data collection, data curation, software, writing - original draft. **Frédéric Frappart:** Conceptualization, supervision, writing - review and editing, project administration, funding acquisition.
